# Case report: Vulval sebaceous carcinoma: a report of two cases and literature review focus on treatment and survival

**DOI:** 10.3389/pore.2023.1611259

**Published:** 2023-06-30

**Authors:** Xiaoxue Wang, Xin Wei

**Affiliations:** ^1^ Department of Obstetrics and Gynecology, Peking Union Medical College Hospital, Chinese Academy of Medical Sciences, Peking Union Medical College, Beijing, China; ^2^ Department of Obstetrics and Gynecology, The Affiliated Changsha Central Hospital, Hengyang Medical School, University of South China, Changsha, China

**Keywords:** extraocular sebaceous carcinoma, vulval sebaceous carcinoma, surgery, adjuvant therapy, prognosis

## Abstract

**Background:** Extraocular sebaceous carcinoma (SC) arising in the vulva is extremely rare that no treatment consensus has been well-defined.

**Case presentation:** We here presented two cases of vulval SC in a 31-year-old and a 62-year-old woman, respectively. Radical wide local excision was performed with free margin and they received no postoperative adjuvant therapy. No evidence of disease was detected after follow-ups for 12 months and 49 months, respectively. A comprehensive literature review of vulval SC was further conducted and other ten cases were included. The mean age was 55.9 years, nine patients were diagnosed with FIGO stage I diseases while the remaining three patients had metastatic lesions at initial diagnosis. Surgery was the mainstay treatment option that 11 (91.7%) underwent surgical resection, of which 5 patients received inguinal lymphadenectomy and 2 patients showed lymph nodes involved. Radiotherapy and chemotherapy were given in 2 and 1 patient, respectively. Two patients experienced recurrence within 1 year after initial therapy. At the final follow-up, ten patients had no evidence of disease, one patient was alive with the disease, and only one died of the disease.

**Conclusion:** Radical wide local excision may be preferred in early-stage vulval SC and utilization of sentinel lymph node sampling should be recommended. Postoperative adjuvant therapy may be spared in patients with negative surgical margin and absence of lymph node involvement. Treatment of vulval SC referring to the guidelines of vulvar cancer should be administered in case of positive margins or metastatic disease.

## Introduction

Sebaceous carcinoma (SC) is an uncommon cutaneous malignant tumor with aggressive potential, accounting for less than 5% of all cutaneous malignancies [1]. SC can be divided into two subtypes, periocular SC and extraocular SC, in which about one-third to up to 75% of the SC are classified as periocular SC [2]. Furthermore, over 90% of the extraocular SC locates in the head/neck region, and only 7.2% originates from other sites [3].

Vulval SC is an extremely rare type of extraocular SC and only a handful of cases have been reported [4]. Characterization of the management of this rarity is completely accumulated through several case reports, and treatment strategies have varied greatly, with some researchers applying radical vulvectomy while others chose wide local excision, and some merely administrated excisional biopsy as primary surgical treatment [4–8]. The necessity of inguinal lymphadenectomy is still controversial [2, 4]. Postoperative chemotherapy and radiotherapy have also been reported but the benefit remained unclear [5, 9, 10]. However, currently, the studies only present narrative descriptive survival outcomes, the detailed information on clinical characteristics and prognosis, especially prognostic predictors, needs to be explored in greater depth.

To investigate the clinical characteristics and outcomes of patients with vulval SC, a retrospective study of 12 patients was conducted, including two patients diagnosed in Changsha Central Hospital and 10 cases reviewed in published research. The recurrence-free survival (RFS) and disease-specific survival (DSS) of these patients were further evaluated.

## Materials and methods

Our two-step study was approved by the Institutional Review Board of the Changsha Central Hospital. Firstly, we performed a pathological review in the database of our hospital and two cases of vulval SC treated in our hospital were identified. Secondly, in order to enroll all the suitable vulval SC patients reported between 1970 and 2022, we used the following keywords to search in major medical databases (PubMed, Embase, Web of Science, and Scopus): “vulval sebaceous carcinoma”; “sebaceous carcinoma of the vulva”; “primary vulval sebaceous carcinoma”; “sebaceous carcinoma of female genital tract”; “extraocular sebaceous carcinoma.” All the related articles of vulval SC cited by the screened papers would have been also evaluated to determine whether they were eligible. Patients who were diagnosed as extraocular sebaceous carcinoma of other sites or secondary vulval sebaceous carcinoma were excluded from final analysis. Similarly, those who lacked either detailed clinical characteristics or survival outcomes were also eliminated. To ensure the scientific nature and reliability, we excluded vulval SC patients from letter to editors and non-English publications. Moreover, unrelated articles, including imaging studies and or pathological investigations of vulval SC were not subjected to analysis. The Supplementary Figure S1 shows the screening process of our research. We eventually included 12 patients with vulval SC, including 10 patients identified in literature review and the two patients treated in our hospital.

After generation of the database, we summarized and analyzed the patients’ clinical characteristics, treatment details, and survival outcomes. In this study, we defined the interval between the date of initial treatment and the confirmation of disease relapse as recurrence-free survival (RFS). Disease-specific survival (DSS) was calculated from the date of diagnosis establishment up to the death caused by tumor or the last follow-up.

### Statistical analysis

Variables descriptions in this study were determined by their distributions. The survival probability was calculated according to the Kaplan-Meier method and the log-rank test. All the statistical analyses were performed by the SPSS (version 27.0; IBM SPSS, Armonk, NY, USA) software.

## Results

### Case presentation

A total of 52 patients with sebaceous carcinoma were included in the screening and 30 of them were female. In this cohort, periocular sebaceous carcinoma (12 cases) was the most predominant subtype, followed by breast (7 cases), scalp (4 cases), nose (3 cases), parotid gland and inguinal region (1 case, each), in descending order. Furthermore, only two patients were diagnosed with sebaceous carcinoma of the vulva, accounting for 6.7% of this female population.

#### Case 1

A 62-year-old woman visited the local hospital due to vulval erythema with erosion and itching for 3 years. A vulval skin biopsy was then performed and pathology could not exclude Paget’s disease. Subsequently, an inter-institutional pathological consultation diagnosed carcinoma of the skin appendage, tending to originate from the sebaceous gland. Further whole-body PET/CT did not reveal lesions in other sites and no enlarged lymph nodes were observed. The immunohistochemical (IHC) staining showed partial expression of AR and CK8/18, CK7 and P63 were also positive, with a Ki-67 proliferative index of approximately 70%. No elevated tumor markers or specific comorbidity was noted. Physical examination showed multiple erythemas with the largest diameter of about 2.5 cm on the surface of the left labium and mons pubis, and the distance to the middle line was over 2 cm.

**FIGURE 1:**
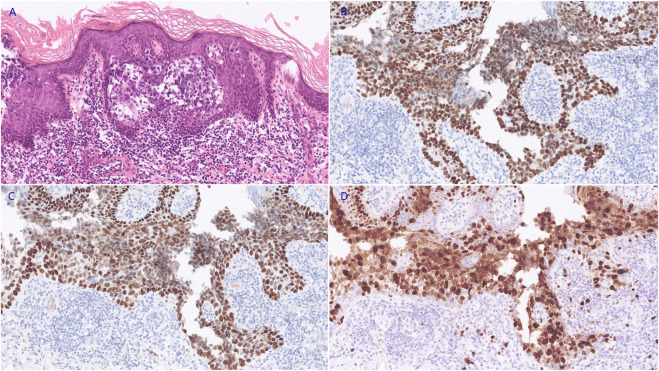
The neoplastic cells demonstrated marked pleomorphism, with increased nucleo-cytoplasmic ratio and irregular nucleus shape (**(A)**, HE staining, 200X). The IHC staining revealed positive expression of P40 (**(B)**, 200X) and P63 (**(C)**, 200X). The Ki-67 proliferative index was approximately 70% (**(D)**, 200X).

Radical wide local excision with a surgical margin of 2 cm and deep to the fascia was performed (Supplementary Figure S2). The final pathology confirmed the diagnosis of sebaceous carcinoma arising in the vulva. The IHC staining showed positive expression of CD15, CD10, P40, and P63, the Ki-67 proliferative index was 70% (Figure 1). The tumor-infiltrating depth was 1 mm without margin involved (>1 cm), and FIGO stage IB was established based on the pathology and preoperative imaging.

The postoperative recovery was uneventful and she was discharged 4 days after the surgery. No adjuvant chemotherapy or radiotherapy was administrated. She was requested to follow up in a 3 months interval and showed no evidence of disease 12 months after the surgery.

#### Case 2

**FIGURE 2:**
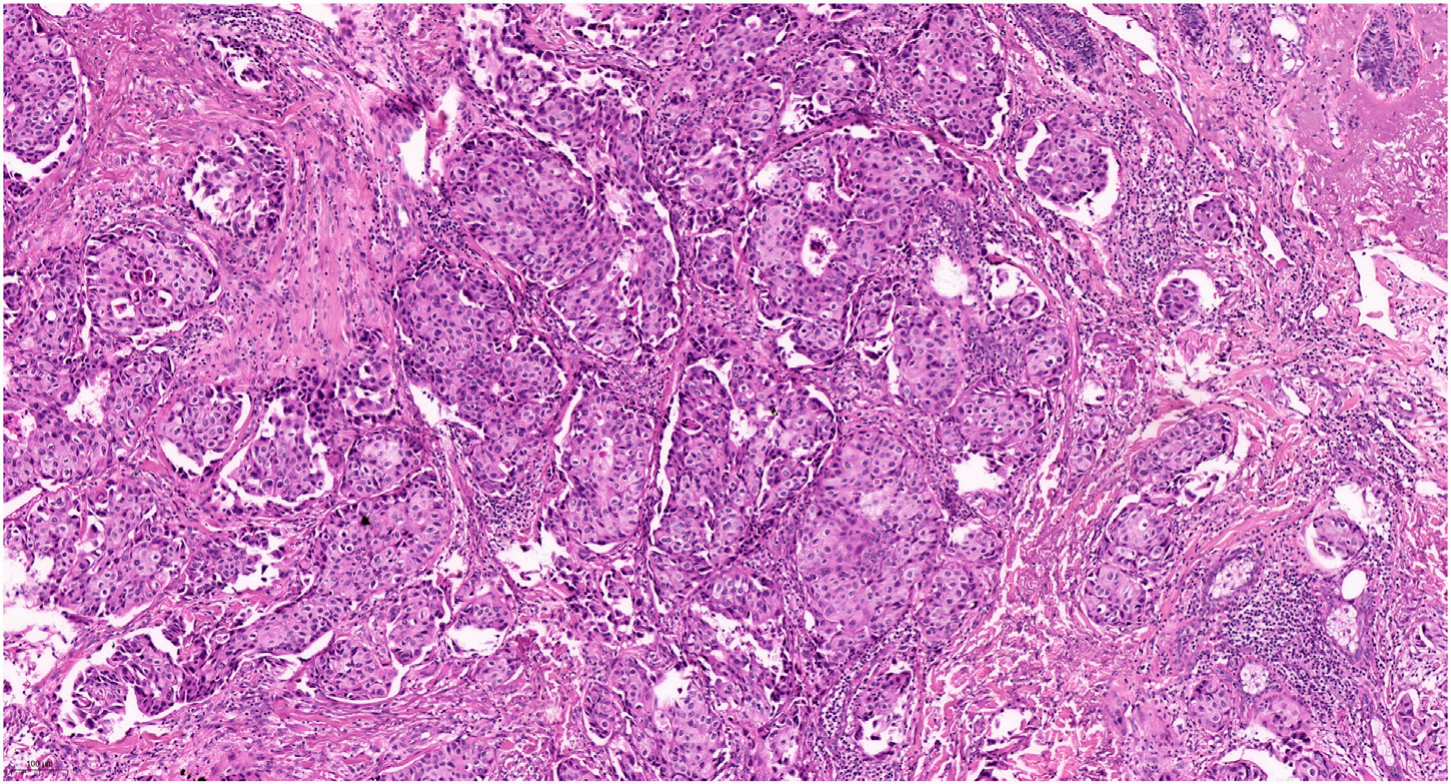
Pathology of the second patient revealed classic morphology of sebaceous carcinoma (HE staining, 100X).

A 31-year-old woman presented a 0.5-cm mild painful nodule with slight itching of the left labium major for nearly 2 years. An excisional biopsy confirmed the diagnosis of moderately differentiated vulval SC (Figure 2). The surgical margin was involved by the tumor and subsequent whole-body PET/CT revealed no metastatic lesion. She was referred to our hospital after the diagnosis had been established. Radical wide local excision was performed. Similarly, we set the surgical margin of 2 cm and deep margin to the fascia, aiming to achieve a pathological margin of at least 8 mm. Postoperatively, pathology revealed no residual tumor, and no further adjuvant therapy was administrated. She remains free of disease at 49 months after treatment.

### Literature review

Twelve patients were included after screening, and the mean age was 55.9 years, only three were younger than 45 years. The mean gross mass size was 1.7 cm in these patients. Most of the primary lesions were in the labia. At the time of initial diagnosis of vulval SC, lesions in 9 patients were classified as FIGO stage I, while metastatic disease was found in three cases, of which two had inguinofemoral lymph nodes involved and one had retroperitoneal lymph nodes and lung metastasis.

The manifestations of vulvar SC were unspecified and may be asymptomatic (Table 1). All patients underwent surgical treatment after diagnosis, except one patient who had lung metastasis. However, the surgical options varied, and excisional biopsy, local excision, wide local excision, radical wide local excision, and radical hemivulvectomy had been used variably. Furthermore, five patients also received lymphadenectomy and two cases showed nodal involvement. Most of these patients (9/11, 81.8%) did not receive any postoperative adjuvant therapy. Radiotherapy was administrated in the two patients who had inguinofemoral lymph node metastasis. Besides, the patient with lung metastasis received platinum-based chemotherapy combined with everolimus but had a poor response.

**TABLE 1:** The clinical characteristics and survival outcomes in patients with vulval SC.

Patients	Age (y)	Manifestations; sites	Sizes (cm)	Surgery	Adjuvant therapy	R1 and treatment	R2 and treatment	Result of follow-up
1	62	Erytherma with erosion and itching; vulva	2.5	Radical wide local excision (Ki-67 70%)	N	N	N	NED at 9 months
2	31	Pain, pruritis nodule; left labium majus	0.5	Radical wide local excision (Ki-67 10%)	N	N	N	NED at 46 months
3 (Rulon, 1974)	31	Asymptomatic; labium minora	2.0*1.1	Simple excision	N	N	N	NED at 13.5y
4 (Kawamoto, 1995)	78	Asymptomatic; labium minora	2.5*1.5	Simple vulvectomy and left inguinal LN	Radiotherapy	N	N	NED at 17 months
5 (Carlson, 1996)	46	Pruritic lesion; labium majus	NA	Left radical hemivulvectomy and left inguinal LN	N	N	N	NED at 31 months
6 (Escalonilla, 1999)	76	Bowenoid papulosis; labium majus	4*3	Left radical hemivulvectomy	N	N	N	NED at 1y
7 (Khan, 2003)	49	Vaginal bleeding; labium majus	0.5	Wide local excision and bilateral inguinal LN	Radiotherapy (50 Gy in 25 fractions)	7 months after surgery, right side of the vulva; excision	4 months after R1, multiple seeding of vulva, perianal region, and right groin; palliative chemotherapy (EPI, 5-Fu, cisplatin)	AWD at 1y
8 (Pusiol, 2011)	51	Asymptomatic; labium majus	2.5*1.5	Hemivulvectomy	N	N	N	NED at 1.5y
9 (Sullivan, 2016)	76	Mild discomfort; vulva	0.5	Wide local excision and left inguinal LN	N	N	N	NED at 10 months
10 (Alharthi, 2021)	27	Micturition; labium majus	1	Biopsy (Ki-67 50%–60%)	Chemotherapy (TC) + everolimus	NA	Initial retroperitoneal lymph nodes and lung metastases	DOD at 8 months
11 (Yamamoto, 2021)	66	Vulval discomfort; labium minora	0.8	Local excision and sentinel LN (Ki-67 50%)	N	N	N	NED at 14 months
12 (Rocha, 2022)	78	Pruritis; labium majus	1.2*1.0	Excision biopsy	N	3 months after surgery at labium majus; excision	2.5 years after R1, labium minora; partial right vulvectomy and right sentinel node biopsy	NED at 3.75y

Abbreviations: N, No; NA, not applicable; LN, lymphadenectomy; TC, taxol plus carboplatin; R1, first recurrence; R2, second recurrence; NED, no evidence of disease; AWD, alive with disease; DOD, die of the disease.

The median follow-up time was 1.3 years (range: 0.7–13.5 years). During the follow-up, two patients experienced recurrence within 1 year after initial therapy, among them one was diagnosed as FIGO stage III (bilateral inguinal lymph nodes metastasis). These two patients underwent surgical excision but both presented a second recurrence. One of them received palliative chemotherapy due to multiple metastatic lesions. Another patient received a partial vulvectomy that achieved no evidence of disease. At the final follow-up, ten patients achieved no evidence of disease, one patient was alive with the disease, and one died of the disease. The 5-year DSS rates was 91.7%, and the 5-year RFS rate was 81.8%. The median RFS and DSS had not been achieved, with a mean RFS and DSS of 11.1 years (8.1–14.1, 95% confidence interval [CI]) and 12.4 years (10.4–14.4, 95% CI), respectively.

## Discussion and conclusion

Our study presents two rare cases of vulval SC and provides a literature review of published cases of vulval SC focusing on clinical characteristics and survival outcomes in this unique population. Patients with vulval SC usually manifested lesions that resemble other vulval cancers of common types without distinct symptoms, and the survival outcome was satisfactory.

The mainstay therapeutic option of extraocular SC remains surgery with complete circumferential peripheral and deep margin assessment to guarantee adequate margins [2]. Rocha et al. [8] reported a patient who experienced two times recurrences due to a surgical margin of less than 1 mm, after a partial vulvectomy with enough margin she achieved no evidence of disease for 1 year. Indeed, the NCCN guidelines recommend re-excision of positive margins or those classified as close (<8 mm) [11]. Our patients are alive without disease relapse after radical wide local excision with a 2-cm margin. Moreover, our study showed 10 of 12 patients showed no evidence of disease at last follow-up, indicating a favorable survival outcome in patients with vulval SC. Furthermore, radical vulvectomy inevitably substantially increases the psychosexual morbidity of the treatment. We herein suggest that radical wide local excision with adequate peripheral and deep margins may be preferred in patients with early-stage vulval SC.

The necessity of inguinal lymphadenectomy has not been well defined. Our study showed a relatively high lymph node involvement rate of 40% in five patients who underwent inguinal lymphadenectomy. Besides, if we include another case reported in a letter [12], this rate goes up to 50% (3 of 6 cases), and whether the lymph node involved or not seemed to have no relation with tumor size, as even a patient with only 5 mm lesion was presented lymph node metastasis. It might be hypothesized that the size of the carcinoma does not equal the lesion site. Nonetheless, only 10 (0.9%) patients had positive lymph nodes in 1,070 cases of extraocular SC [13]. It is unclear why the vulval SC presents a considerably high regional lymph node involvement rate, maybe due to the profuse lymph circulation of the perineum and the location at the vulva may be potentially an intrinsic risk factor for tumor metastasis. Furthermore, the relation between disease-specific mortality of vulval SC and regional lymph node involvement is unknown. To avoid the omitting of positive lymph node excision and reduce the side effect of lymphadenectomy, sentinel lymph node (SLN) sampling has been increasing utilized in vulval SC and extraocular SC [2, 4]. In the GROINSS-V study, the recurrence rate was only 2.3% in 403 patients with negative SLN sampling of vulvar carcinomas, with a 3-year DSS of 97% and remarkably reduced surgical morbidity [14]. Hence, comprehensive imaging, preoperative evaluation and routine SLN sampling in early stage vulval SC may be practical. However, in cases of macrometastases in SLN, bilateral inguinal lymphadenectomy should be conducted since inguinofemoral radiotherapy without surgical resection had a significantly higher recurrence rate [15].

The utility of postoperative adjuvant therapy remains controversial. Radiotherapy may be a candidate for monotherapy for extraocular SC patients who are medically inoperable or have surgically unresectable tumors, positive margin, or nodal metastases with a recommended dose of 50–70 Gy [1, 2]. The GROINSS-V-II trial found that inguinofemoral radiotherapy is a safe alternative for inguinofemoral lymphadenectomy in patients with sentinel node micrometastases [15]. Three patients underwent surgery with radiotherapy due to inguinal lymph node metastasis, all of them were alive and two of them were disease-free [5, 9, 12]. Therefore, postoperative radiotherapy or chemotherapy referenced in guidelines of extraocular SC or vulval cancer may be reasonable. Nonetheless, the only death related to vulval SC in our cohort that received platinum-based chemotherapy due to lung metastasis showed a poor response and succumbed to the disease only 8 months after diagnosis [10].

It should be emphasized that screening of Muir-Torre syndrome (MTS) in patients with vulval SC must also be performed. MTS is characterized by the presence of sebaceous neoplasms and one or more visceral malignancies [16]. The sebaceous tumors include solitary or multiple sebaceous adenoma and/or carcinoma, and colorectal cancer and endometrial cancer are the two most common visceral malignant tumors [17]. Moreover, some MTS patients also have germline mutations in DNA mismatch repair genes MLH1 or MSH2, and are considered a subtype of Lynch syndrome [18]. In our study, both patients had PET/CT to screen for other lesions and coexisting tumors. Although the incidence of MTS is extremely rare [19], realizing the probability in vulval SC patients may allow appropriate screening for visceral malignant tumors to reduce morbidity and mortality.

To sum up, the survival outcomes in patients with vulval SC were favorable with a 5-year RFS rate of 81.8% and a 5-year DSS rate of 91.7%, respectively. Radical wide local excision may be preferred in early-stage vulval SC and utilization of SLN sampling should be recommended. Postoperative adjuvant therapy may be spared in patients with negative surgical margin and absence of lymph node involvement. Treatment of vulval SC referring to the guidelines of vulvar cancer should be administered in case of positive margins or metastatic diseases. Moreover, screening of MTS in patients with vulval SC should be emphasized, especially in young patients. However, due to the extremely rarity of vulval SC, further research is warranted.

## Data Availability

All data generated or analyzed during this study are included in this published article and the Supplementary Information Files. The datasets used and/or analyzed during the current study can be obtained from the corresponding author upon reasonable request.

## Supplementary material

SUPPLEMENTARY FIGURE S1 The screening details of literature review in our study.

SUPPLEMENTARY FIGURE S2 The surgical excision specimen of case 1 (suspicious lesions were marked with suture).
