# Supplementary figures and images for: Case report: Vulval sebaceous carcinoma: a report of two cases and literature review focus on treatment and survival
Source: Pathol Oncol Res. 2023 Jun 30;29:1611259. doi: 10.3389/pore.2023.1611259 (PMC10345201; doi:10.3389/pore.2023.1611259)

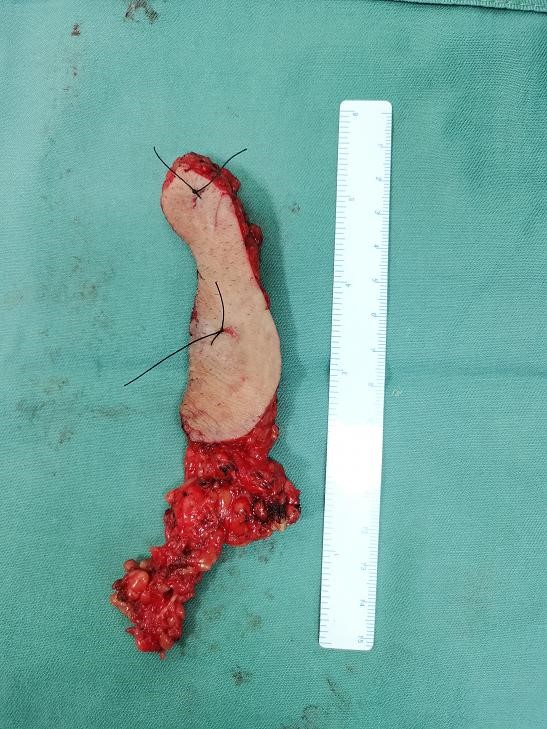

Supplement: Supplementary file 1 [file Image1.JPEG]

Figure S1. The detailed inclusion process according to the PRISMA flow diagram in our study.

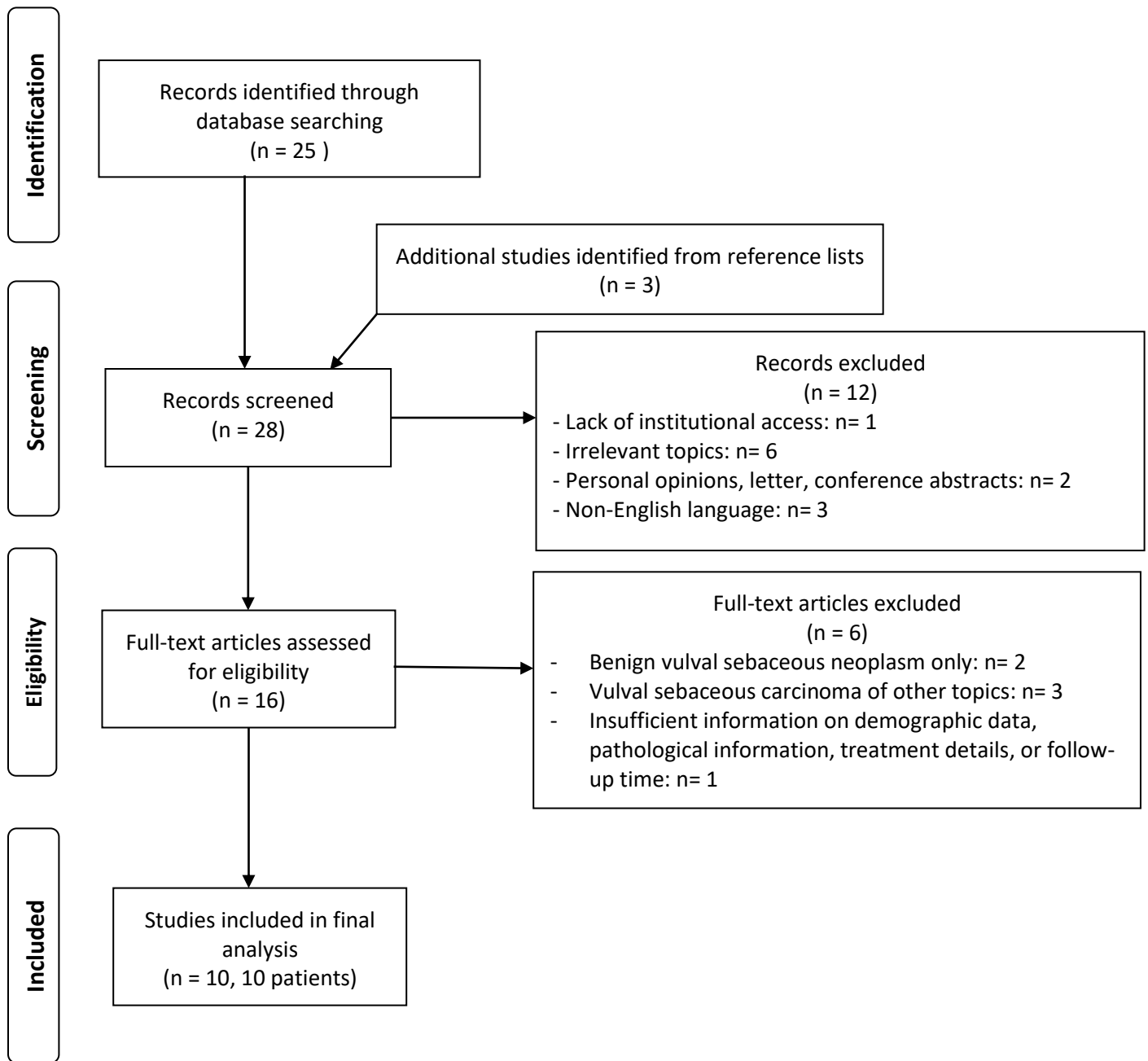

Supplement: Supplementary file 2 [file DataSheet1.PDF]
